# Rate of decline in residual kidney function and cognitive impairment in incident haemodialysis patients: A prospective, longitudinal analysis of the BISTRO trial cohort

**DOI:** 10.1371/journal.pone.0349109

**Published:** 2026-06-08

**Authors:** Kerry-Lee Rosenberg, Indranil Dasgupta, Dorothea Nitsch, John Belcher, Ken Farrington, Simon Davies

**Affiliations:** 1 Centre for Kidney and Bladder Health, University College London, London, United Kingdom; 2 London School of Hygiene and Tropical Medicine, London, United Kingdom; 3 University Hospitals Birmingham NHS Foundation Trust, Birmingham, United Kingdom; 4 School of Health Sciences, University of Birmingham, Birmingham, United Kingdom; 5 School of Medicine, Faculty of Medicine and Health Sciences, Keele University, Newcastle, United Kingdom; 6 Renal Medicine, East & North Hertfordshire NHS Trust, Hertfordshire, United Kingdom; Saint-Joseph University of Beirut, LEBANON

## Abstract

**Background:**

Previous studies suggest loss of residual kidney function (RKF) on haemodialysis is associated with cognitive impairment (CI). This study investigated the association of rate of change in RKF with risk of new CI and change in cognition after haemodialysis initiation and the association between dialysis treatment parameters and changes in cognition.

**Methods:**

This prospective cohort study used data from the Bioimpedance Spectroscopy to Maintain Renal Output trial (BISTRO). Cognition was assessed using the Montreal Cognitive Assessment (MoCA). Logistic regression and generalised estimating equations were used to estimate the association of rate of decline in RKF and clinical parameters with risk of CI at 12 and 24 months after haemodialysis start and mean change in MoCA score per year.

**Results:**

The study included 435 participants. Rate of decline in RKF was not associated with risk of CI at 12 or 24 months or with mean change in MoCA score. Higher dialysate temperature was associated with a mean annual improvement in MoCA score of 1.12 per one degree Celsius (95% CI 0.23 to 2.01). Amongst those without left ventricular failure, an interdialytic weight gain ≥2 kg was associated with an annual increase in MoCA score of 1.62 (95% CI 0.21 to 3.04), compared to those with weight gains ≤0.5 kg. Conversely, a weight gain ≥2 kg was associated with a worsening MoCA score of −3.08 (95% CI −5.76 to −0.40) amongst those with left ventricular failure (p-value for interaction < 0.001).

**Conclusions:**

We found no association between rate of decline in RKF and cognitive performance. These findings may reflect selection bias or the effect of relatively slow rate of decline in RKF. Higher interdialytic weight gains were associated with worsening cognition amongst those with left ventricular failure, underscoring another challenge of fluid management in this group of patients.

## Introduction

Chronic kidney disease (CKD) is an independent risk factor for cognitive impairment (CI) [1,2]; defined as a decline from previous performance in one or more cognitive domains, such as language, executive function, attention or memory [3]. CI is particularly common amongst those with end-stage kidney disease (ESKD) receiving haemodialysis, with point prevalence estimated at 22–77% in this population [4–8]. CI adds to an already high burden of morbidity amongst haemodialysis patients and has been associated with poor outcomes, including all-cause mortality and hospitalisation [9,10]. CI, therefore, poses an important clinical challenge and identifying potentially modifiable risk factors will become increasingly relevant as the prevalent ESKD population grows and ages.

The aetiology of CI in CKD is multifactorial. Cerebrovascular disease, including microvascular injury [11–13], accumulation of metabolites, oxidative stress, anaemia, vitamin D deficiency and polypharmacy are all likely contributors [14,15]. In addition, there is evidence to suggest that factors specific to haemodialysis treatment may be associated with incidence and progression of CI [15–20]. The impact of haemodialysis on cognition is likely to be related both to adequacy of the treatment and its cardiovascular consequences [14,15]. Clearance of medium-sized metabolites [14,21], fluid shifts resulting in intradialytic hypotension [22] and reduction in cerebrovascular blood flow during treatment (even without symptomatic hypotension) [23,24] have all been implicated. In addition, it has been hypothesised that loss of residual kidney function (RKF) may contribute to CI in dialysis populations [25–27]. Possible causal mechanisms for this association include increased clearance of uraemic toxins in those with preserved RKF and improved fluid balance and haemodynamic stability. These studies are limited by their cross-sectional design and only one included a haemodialysis population [27]. It is also possible that factors related specifically to the efficacy and safety of haemodialysis treatment may contribute to risk of CI. Reduced dialysate temperature [28] has been associated with reduced white matter ischaemic injury on brain imaging and use of haemodiafiltration has been associated with higher verbal fluency scores and cerebral blood flow [29,30]. Conversely, it has been hypothesised that fluid overload is associated with increased risk of CI [31].

There is a paucity of data examining the association between RKF and CI in haemodialysis populations. In particular, longitudinal analyses which capture change in cognition over time are lacking. Secondly, the association between clinical markers of volume status and treatment parameters, designed to reduce haemodynamic shifts and improve clearance, with CI is poorly understood.

This study aims, firstly, to examine the association of rate of decline in RKF with risk of CI and change in cognition amongst incident haemodialysis patients. Secondly, it aims to investigate the association of modifiable, dialysis treatment-specific factors, including treatment parameters and markers of fluid status, with change in cognition in this population.

## Methods

This is a prospective cohort study of participants in the Bioimpedance to Maintain Renal Output (BISTRO) study [32], a multi-centre, randomised controlled trial. BISTRO aimed to investigate the effect of using of bioimpedance (BI) in establishing patients’ normally hydrated weight on rate of decline in RKF amongst incident haemodialysis patients. The primary endpoint was time to anuria and no difference between intervention and control groups was found [33]. This study, therefore, treats the sample as a single cohort.

Local ethical approval was obtained from the London School of Hygiene and Tropical Medicine Research Ethics Committee (reference number 30325). The BISTRO trial had UK Integrated Research Ethics approval (206213). The “Strengthening the reporting of observational studies in epidemiology” (STROBE) guidelines [34] were used in the preparation of this report (S1 Table).

### Setting and participants

Recruitment to the trial and baseline data collection took place over a 29 month period (17/04/2017–30/09/2019) in 34 dialysis units across the United Kingdom (UK). The trial was completed on 31/07/2023.

Potential participants were identified using local processes in each unit [33] and written consent was obtained. Adult patients (> 18 years of age) who were within three months of commencing centre-based, maintenance haemodialysis for ESKD were eligible for inclusion. Those who had not yet started dialysis required a urine output of > 500 ml/day or a mean urea and creatinine clearance of >3ml/min/1.73m^2^ (measured by a 24 hour urine collection). Those already established on dialysis required a urine output of >500ml in the interdialytic period or a mean urea and creatinine clearance of >3ml/min/1.73m^2^ as measured by an inter-dialytic urine collection and pre and post-dialysis blood tests [32,35]. Full inclusion and exclusion criteria have been described previously [32].

### Data collection and follow-up

Participants completed a baseline study visit at dialysis start and were followed up for 24 months or until death, transplantation, withdrawal of dialysis or patient choice to leave the study [32]. At the baseline visit characteristics, including age, sex, ethnicity and comorbidities (including presence of previously diagnosed diabetes, ischaemic heart disease, left ventricular failure and peripheral vascular disease), were recorded. Comorbidities were defined as a pre-existing diagnosis listed in the clinical record. They are categorised into domains derived from the externally validated Stoke Comorbidity Score [36,37]. In this system pre-existing cerebrovascular disease is included within the peripheral vascular disease domain. Participants completed a baseline cognitive assessment and RKF was calculated.

Follow up visits were scheduled monthly for the first three months, then bimonthly for the following three months and every three months thereafter. Participants underwent a clinical fluid balance assessment, performed by the clinician and recorded on a study proforma, at each visit. Incidence of intradialytic hypotension was recorded as part of these assessments. RKF was measured at each follow up visit. Cognitive assessment was repeated at the 12 and 24 month visits. Cognitive assessments were performed pre-dialysis or within the first 30 minutes of treatment. The trial protocol, including patient participation and involvement, is described in detail elsewhere [32].

### Variables

Cognition (outcome variable) was assessed using the Montreal Cognitive assessment (MoCA). This tool provides a global assessment of cognition, including executive function. The MoCA has been previously validated against a cognitive battery in haemodialysis populations and found to be sensitive for detection of mild CI; with a suggested cut-off score of 23.5 to 24 out of 30 [38,39]. This study will, therefore, define CI as MoCA score less than 24.

Glomerular filtration rate (GFR) was used to measure RKF and was calculated using a, validated, study-specific standard operating procedure [32,35]. In this study rate of decline in RKF (primary exposure of interest) was measured in ml/min/1.73m^2^/month and was calculated as the difference between measured GFR and baseline, divided by number of months between measurements.

Secondary exposures were included as follows. Presence of intradialytic hypotension was defined as symptomatic intradialytic hypotension as recorded in patients’ dialysis records at any point in the follow up period (binary). Inter-dialytic weight gain (IDWG) was calculated as the difference between pre and post dialysis weight at each follow-up visit and a mean value for all recorded IDWGs was obtained for each participant. A mean pre-dialysis diastolic and systolic blood pressure were calculated for each participant, encompassing total follow up time and all measurements taken. Dialysate temperature was a centre-level variable and represents the default temperature setting used in each unit.

### Outcomes

The primary outcomes of the study were:

- Association of rate of decline in RKF with risk of CI, defined as MoCA score < 24, at 12 and 24 months after initiation of haemodialysis.
- Association of rate of decline in RKF with mean change in MoCA score per year after initiation of haemodialysis.

The secondary outcomes of the study were

- Association of clinical parameters (i.e., treatment type, mean IDWG, mean pre-dialysis blood pressure, presence of intradialytic hypotension and dialysate temperature) with mean change in MoCA score per year after initiation of haemodialysis.

### Statistical analysis

Data was analysed using Stata 18^TM^. A 5% significance level was applied throughout.

Demographic and clinical characteristics of the cohort were summarised according to baseline cognitive status. Characteristics were compared between groups using analysis of variance for continuous variables with normal distribution, Mann Whitney rank sum tests for continuous variables not normally distributed and chi-squared tests for categorical variables.

Logistic regression models were used to examine the association of rate of decline in RKF with risk of CI at 12 months and 24 months after start of dialysis. Separate analyses were carried out for each timepoint. Participants with MoCA score < 24 (i.e., with CI) at baseline were excluded from these models and a complete case analysis was used. Age and sex were included in the models a priori. Other exposures, including ethnicity and comorbidities, were added to the model in turn in order to assess for confounding. Results were reported as crude and adjusted odds ratios, with 95% confidence intervals.

A generalised estimating equation was used to investigate the association of rate of decline in RKF with mean change in MoCA score over the first 12–24 months of haemodialysis treatment. The dependent variable in this model was change in MoCA score from baseline to 12 months or from 12 to 24 months, treated as a continuous variable. The exposure of interest was rate of change in RKF from baseline to 12 or 24 months, measured in ml/min/1.73m^2^/month, also included as a continuous variable. Age and sex were included in the model *a priori*. Ethnicity and comorbidities were added to the model in turn in order to assess for confounding effect. Results were reported as mean change in MoCA score per one unit increment in the parameter of interest and a 95% confidence interval.

A sensitivity analysis was carried out for the primary outcomes, using multiple imputation in order to account for loss to follow up. Observations were not imputed for those who died or were transplanted. The imputation model included age, sex, ethnicity, comorbidities, RKF and MoCA score.

Generalised estimating equations were used to investigate the effect of clinical parameters on mean change in MoCA score from baseline to 12 months and from 12 to 24 months after initiation of haemodialysis. The dependent variable (change in MoCA score) was modelled as a continuous variable. Models were adjusted a priori for age and sex. The exposures of interest (mean IDWG, mean pre-dialysis systolic and diastolic blood pressures, presence of intradialytic hypotension, treatment type and dialysate temperature) were each added to the model in turn. Exposures of interest were initially modelled separately to assess for confounding by ethnicity or comorbidities. Interaction terms were added to each model in turn, in order to assess for effect modification by comorbidities.

## Results

**Fig 1:**
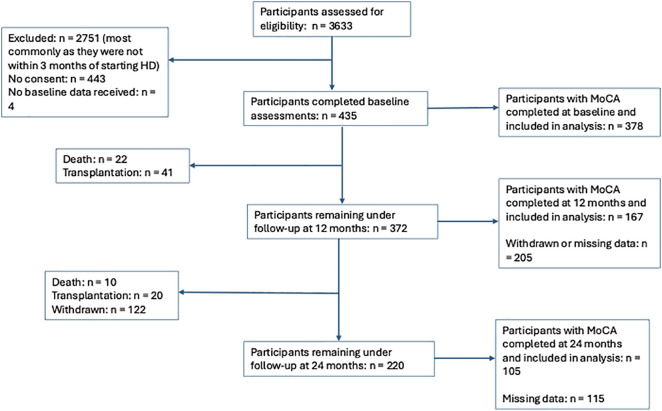
Flow diagram illustrating entry of participants into the study, follow-up and those included in the analysis.

The cohort included 435 participants. Recruitment to the BISTRO study, including the number of exclusions and withdrawals, has been described in detail previously and is demonstrated in Fig 1 [33,40].

Baseline characteristics of the participants are shown in Table 1. Mean age was 61.5 years (SD 14.1). Of the cohort, 70.3% were male and 79.8% were white. Mean baseline GFR was 4.8 ml/min/1.73m^2^ (SD 2.8) and mean change in GFR over 24 months of follow up was −0.09 ml/min/1.73m^2^/month (95% confidence interval – 0.10 to −0.08). The trial endpoint of anuria was met by 72 participants (16.6%) and follow up for patient reported outcomes continued in this group. In total, 122 participants withdrew from the trial (28%), 32 (7.4%) died and 61 (14.0%) received a kidney transplant. Median follow up was 13 months (IQR 5–24 months).

**Table 1:** Baseline characteristics of participants, overall and by Montreal Cognitive Assessment (MoCA) score < 24 and ≥ 24.

	All (n = 435)	Cognitive Impairment (MoCA < 24) (n = 110)	No Cognitive Impairment (MoCA ≥ 24) (n = 268)	P – value^1^
Age (years) at study entry, mean (SD)	61.5 (14.1)	65.8 (13.2)	59.3 (14.4)	**< 0.001**
Sex, n (%)				
Male	306 (70.3)	71 (64.6))	196 (73.1)	0.122
Female	128 (29.5)	38 (34.6)	72 (26.9)	
Missing	1 (0.2)	1 (0.9)		
Ethnicity, n (%)				
White	347 (79.8)	79 (71.8)	229 (85.5)	**0.01**
Black/Black British	6 (1.4)	2 (1.8)	2 (0.75)	
Asian/Asian British	9 (2.1)	2 (1.8)	6 (2.24)	
Other	73 (16.8)	27 (24.6)	31 (11.6)	
Comorbidities^2^, n (%)				
Diabetes Mellitus	198 (45.5)	51 (46.4)	120 (44.8)	0.778
Ischaemic heart disease	88 (20.2)	19 (17.3)	54 (20.6)	0.520
Peripheral vascular disease	52 (12)	11 (10)	35 (13.1)	0.409
Left ventricular heart failure	56 (12.9)	16 (14.6)	34 (12.7)	0.628
Treatment type, n (%)				
Haemodialysis	295 (67.8)	67 (60.9)	196 (73.1)	**0.024**
Haemodiafiltration	139 (32)	42 (38.2)	72 (26.9)	
Missing	1 (0.2)	1 (0.9)		
Baseline pre-dialysis blood pressure (mmHg), mean (SD)	**n = 416**	**n = 105**	**n = 260**	
Systolic	150.4 (25.2)	149.1 (25.7)	151.7 (25.5)	0.385
Diastolic	76.3 (15.5)	73.7 (14.6)	78 (15.5)	**0.015**
Dialysate temperature (degrees Celsius), n (%)				
< 36	65 (14.9)	9 (8.2)	45 (16.8)	**0.018**
≥ 36	303 (69.7)	86 (78.2)	175 (65.3)	
Missing	67 (15.4)	15 (13.6)	48 (17.9)	
Baseline measured GFR (ml/min/1.73m^2^), mean (SD)	n = 389	n = 95	n = 244	
	4.8 (2.8)	4.8 (2.8)	4.9 (2.9)	0.770
Mean interdialytic weight gain (kg), n (%)				
< 1.0	134 (30.8)	41 (37.3)	82 (30.6)	0.122
1.0 - 2.0	194 (44.6)	50 (45.5)	114 (42.5)	
>2.0	107 (24.6)	19 (17.3)	72 (26.9)	
Mean interdialytic weight gain (kg), median (IQR)	1.3 (0.5, 2)	1.1 (0.4, 1.8)	1.3 (0.4, 2.0)	0.1784
Presence of intradialytic hypotension^3^, n (%)				
Yes	59 (13.6)	15 (13.6)	38 (14.1)	0.838
No	367 (84.4)	95 (86.4)	225 (84.0)	
Missing	9 (2.0)	0	5 (1.9)	

^1^Comparing group with cognitive impairment to those without. P-values calculated using a One-way ANOVA for continuous variables with normal distribution, Mann Whitney rank sum test for continuous variables not normally distributed and chi-squared test for categorical variables.

^2^Comorbidities defined as a pre-existing diagnosis listed in the clinical record and classified according to the Stoke comorbidity score.

^3^Intradialytic hypotension defined as symptomatic intradialytic hypotension as recorded in patients’ dialysis records at any point in the follow up period (binary).

**Fig 2:**
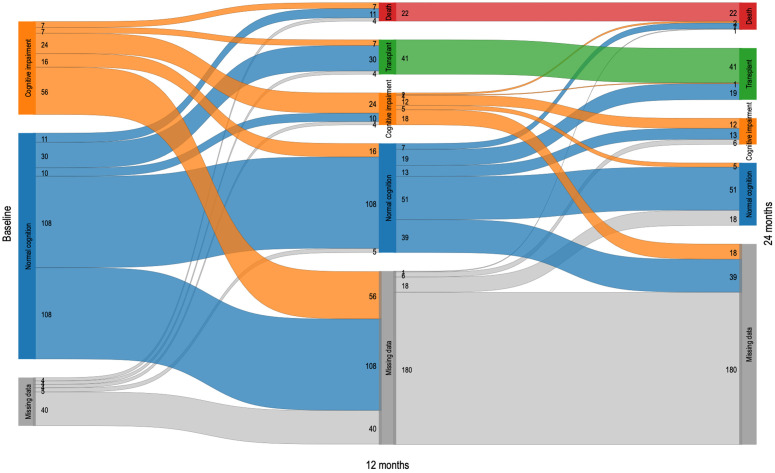
Sankey diagram showing cognition status and death and transplantation at baseline and 12 and 24 months after dialysis initiation.

Mean MoCA scores at baseline, 12 and 24 months, as well as prevalence and incidence of CI at each of these timepoints are summarised in Table 2. The number of missing observations are also shown. Baseline characteristics were similar between those with improved, stable and worsening cognition scores (S2 and S3 Tables). Fig 2 illustrates change in cognition status at each timepoint, as well as death and transplantation. After excluding those who died or received a transplant, the group with missing follow up MoCA scores at 24 months were compared with those with complete data. Mean baseline MoCA score was higher in the group with complete data (25.9 versus 24.4, p = 0.011) and the proportion with baseline CI was lower (22.8% versus 36.6%, p = 0.016).

**Table 2:** Montreal Cognitive Assessment (MoCA) scores and prevalence of cognitive impairment at dialysis start and after 12 and 24 months of treatment.

	Baseline	12 months	24 months
**MoCA score, mean (SD)**	25.2 (4.4)	25.1 (4.3)	25 (4.0)
**Cognitive impairment n, (%)**	110 (29.1)	38 (22.8)	31 (29.5)
**New cognitive impairment** ^**1**^ **, n (%)**	–	10 (6.0)	11 (10.5)
**Improved cognition** ^**2**^ **, n (%)**	–	16 (9.6)	12 (11.4)
**n**	378	167	105
**Missing observations**	57	267	329
**Causes of missing data, n (% of missing observations)**			
Transplantation	–	–	61 (18.5)
Death	–	–	32 (9.7)
Withdrawal from trial	–	–	122 (37.1)
Unknown	–	–	114 (34.7)

^1^New cognitive impairment defined as MoCA < 24 if MoCA previously recorded and normal (24 or more).

^2^Improved cognition defined as those scoring 24 or higher (i.e., normal), who had previously scored below 24.

### Primary outcomes

In logistic regression models adjusted for age and sex, rate of decline in RKF was not associated with risk of CI at 12 months or 24 months after start of haemodialysis (Table 3). No confounding effect by ethnicity or comorbidities was demonstrated at the 12 or 24 month timepoints.

**Table 3:** Results of logistic regression models for association of rate of decline in residual kidney function with cognitive impairment (MoCA^1^ < 24) at 12 and 24 months after start of haemodialysis^2^.

	Unadjusted odds ratio at 12 months (n = 114)	Odds ratio at 12 months; adjusted for age and sex n = 114)	Unadjusted odds ratio at 24 months (n = 78)	Odds ratio at 24 months; adjusted for age and sex (n = 78)
**Rate of decline in eGFR (per 1 ml/min/1.72m** ^**2**^ **/month increase)**	0.64 (0.13–3.18)	0.56 (0.10–3.04)	0.50 (0.02–10.14)	0.64 (0.03–12.97)
**Age (per 1 year increase)**	–	1.01 (0.96–1.07)	–	1.03 (0.98–1.08)
**Female sex (baseline male sex)**	–	2.26 (0.53–9.58)	–	1.95 (0.56–6.80)

^1^Montreal Cognitive Assessment.

^2^Results presented as odds ratio (95% confidence interval).

The results of a generalised estimating equation for mean change in MoCA score following start of dialysis are shown in Table 4. In a mutually adjusted model, no association between time on dialysis (per year) or rate of decline in RKF with change in MoCA score was demonstrated. There was no confounding by ethnicity, diabetes, ischaemic heart disease or peripheral vascular disease.

**Table 4:** Association of rate of decline in residual kidney function with mean change in MoCA^1^ score at 12 and 24 months after start of haemodialysis (n = 182)^2^.

	Unadjusted mean change in MoCA score	Mean change in MoCA score; adjusted for age and sex
**Time (per year from start of haemodialysis)**	−0.25 (−1.36 to 0.87)	−0.25 (−1.37 to 0.87)
**Increase in eGFR (per ml/min/1.73m** ^**2**^ **/month)**	0.20 (−1.61 to 2.02)	0.33 (−1.54 to 2.22)
**Age at start of haemodialysis (per year)**	–	−0.02 (−0.05 to 0.08)
**Female sex**	–	−0.16 (−0.97 to 0.65)

^1^Montreal Cognitive Assessment.

^2^Results presented as mean 1 year change in MoCA score (95% confidence interval).

Given that there was some missing data, both of these analyses were repeated using multiple imputation as a sensitivity analysis. MoCA scores at 12 and 24 months were imputed only for those who did not die or receive a transplant and 50 imputations were run. This resulted in increased sample sizes in all models but no association between rate of decline in RKF and either risk of CI or mean change in MoCA score was demonstrated (S4 and S5 Tables).

### Secondary outcomes

After adjustment for time on dialysis, age and sex, there was no association between treatment type (haemodialysis versus haemodiafiltration), mean pre-dialysis diastolic or systolic blood pressure or presence of intradialytic hypotension and change in MoCA score. A one degree increase in dialysate temperature was associated with a mean increase in MoCA score of 1.12 (95% confidence interval 0.23 to 2.01) per year. A one-kilogram increase in IDWG was associated with a mean increase in MoCA change-score of 0.30 (95% confidence interval 0.15 to 0.46) (Table 5). There was no confounding by ethnicity or comorbidities.

**Table 5:** Association of dialysate temperature and mean interdialytic weight gain with 1-year change in MoCA^1^ score at 12 and 24 months after start of haemodialysis^2^.

	Mean change in MoCA score; adjusted for age, sex and dialysate temperature (n = 136)	Mean change in MoCA score; adjusted for age, sex and mean interdialytic weight gain (n = 152)
**Time (per year from start of haemodialysis)**	−0.44 (−1.55 to 0.67)	−0.23 (−1.36 to 0.93)
**Age at start of haemodialysis (per year)**	0.00 (−0.03 to 0.03)	−0.02 (−0.06 to 0.02)
**Female sex**	0.69 (−0.20 to 1.59)	−0.22 (−1.44 to 1.00)
**Dialysate temperature (per 1 degrees Celsius)**	**1.12 (0.23** to **2.01)**	–
**Mean interdialytic weight gain (per kilogram)**	–	**0.30 (0.15** to **0.46)**

^1^Montreal Cognitive Assessment.

^2^Results presented as mean change in MoCA score (95% confidence interval).

The association of dialysate temperature with change in MoCA score was not modified by ethnicity or comorbidities. However, the association of IDWG with change in MoCA score was modified by presence of left ventricular failure (Table 6). Amongst those without left ventricular failure at baseline, increasing weight gains were associated with an increase in MoCA change-score compared to the group gaining less than 0.5 kg. Conversely, amongst those with known left ventricular failure at baseline, increasing interdialytic weight gain was associated with a decrease in MoCA change-score. In this group, a weight gain of 2 kg or more was associated with a mean change in MoCA of −3.08 (95% confidence interval −5.76 to −0.40), compared to those with weight gain less than 0.5 kg.

**Table 6:** Effect modification of the association of interdialytic weight gain with 1-year change in MoCA^1^ score at 12 and 24 months after start of haemodialysis by left ventricular failure^2^ (n = 181)^3^.

	Fully adjusted model for mean change in MoCA score, including an interaction term for left ventricular failure	
**Time (per year from start of haemodialysis)**	−0.18 (−1.29 to 0.93)	
**Age at start of haemodialysis (per year)**	−0.02 (−0.04 to 0.01)	
**Female sex**	−0.15 (−0.89 to 0.60)	
**Mean interdialytic weight gain in kilograms (baseline <0.5 kg)**	**Left ventricular failure (n = 18)**	**No left ventricular failure (n = 163)**
0.5–0.99	−0.90 (−2.78 to 0.98)	**1.63 (0.13 to 3.12)**
1.0–1.49	**−2.52 (−4.12 to −0.90)**	1.19 (−0.22 to 2.61)
1.5–1.99	−1.44 (−4.31 to 1.42)	**1.68 (0.31 to 3.10)**
>2.0	**−3.08 (−5.76 to −0.40)**	**1.62 (0.21 to 3.04)**

^1^Montreal Cognitive Assessment.

^2^Results presented as mean change in MoCA score (95% confidence interval).

^3^Wald test for effect modification, p < 0.001.

## Discussion

CI was common in this cohort of incident haemodialysis patients, with a prevalence of 29.1% at baseline. The number of participants with new CI at the 12 and 24 month timepoints was similar to the number with improvement in cognition, suggesting a benefit to dialysis initiation in at least some participants and highlighting the complexity of the problem. We did not find evidence for rate of decline in RKF being associated with risk of developing CI at 12 and 24 months after start of maintenance dialysis treatment, nor with mean change in MoCA score. Higher dialysate temperatures were associated with an increase in the mean MoCA change-score over time. In addition, for those without left ventricular failure, a mean interdialytic weight gain of 2 kg or more was associated with an increase in MoCA score of 1.62. Conversely, amongst those with left ventricular failure, a mean weight gain of greater than 2 kg was associated with a worsening in MoCA of −3.08.

This study included a large cohort of incident haemodialysis patients and benefits from its longitudinal design, which allowed regular and precise measurements of RKF over a 24 month follow up period. It is also one of few studies to evaluate change in cognition over time in haemodialysis and it used a cognitive assessment tool which has been validated for use in this population.

There are, however, some limitations. There were 32 (7.2%) deaths during the trial and, of the 122 (27.7%) patients who withdrew, worsening health was the primary reason in 25. A further 47 declined to give a reason and 16 cited inability or unwillingness to comply with trial procedures as the reason for dropping out [33,40], although 51 patients were transplanted which might dilute this to some extent. Secondly, whilst the overall sample size is large (even after accounting for drop-out) there was a significant amount of missing data, exacerbated by the impact of COVID-19 on trial procedures. Investigators were instructed to encourage the completion of patient reported outcome measures but not be forceful, and unwillingness to complete them was not a reason for trial withdrawal by the research nurses. This particularly affected MoCA scores at the 12 and 24 month timepoints, resulting in a loss of power. The logistic regression models at 24 months included only 78 participants, resulting in broad confidence intervals. Participants with CI may be more likely to have a missing MoCA result than those with normal cognition (for example, because they had more difficulty complying with prolonged assessments or were more likely to decline a cognitive test). Our results demonstrate some evidence of this as the group with missing data at 24 months had both a lower mean baseline MoCA score and a higher proportion of CI at baseline compared to those with complete data. Sensitivity analyses using multiple imputation suggest that our results are robust to this loss to follow-up/drop out; assuming that amongst the participants who completed follow-up there are some who represent the likely outcomes of those who did not. However, estimates derived from multiple imputation may be biased if data is missing not at random and that possibility should be considered a limitation of this sensitivity analysis. It was not possible to adjust MoCA scores for educational attainment, which may have resulted in misclassification of the outcome and reduces comparability between individuals. Finally, we acknowledge that the MoCA is a screening tool and is not diagnostic of CI.

Despite suggestive evidence of a relationship, our analysis did not demonstrate an association between rate of decline in RKF with risk of CI or change in MoCA score. A previous cross-sectional study reported that preservation of RKF was associated with higher MoCA scores amongst 78 haemodialysis patients (mean score 27 versus 24), but was unable to examine the effect of change in RKF over time [27]. Two previous studies have demonstrated decline in cognitive test scores over time in haemodialysis patients [16,17]. One of these is limited by the inclusion of only 12 haemodialysis and six peritoneal dialysis patients. Median duration on dialysis at baseline was 54 months in this study [17]. Similarly, the cohort in the second study had a mean dialysis vintage of over two years at baseline [16]. It is difficult to draw comparisons between these studies and our incident haemodialysis cohort. However, their findings may suggest that an extended follow up time is required in order to observe changes in cognition in incident dialysis populations, especially as residual kidney function was relatively well preserved in BISTRO and its preservation during the first two years was associated with better survival at 5 years [41].

Whilst this study may further our understanding of the association between change in RKF and cognitive performance, the impact of selection bias should be considered and the findings must be interpreted with caution. It is possible that the sample analysed includes a group of relatively fit dialysis patients, with greater cognitive reserve and less frailty (given the rigours of the trial protocol); and who are less vulnerable to the effects of loss of RKF over time than a more comorbid real-world cohort may be. To caveat this, however, data suggests that amongst older populations reaching ESKD the number of cognitively vulnerable patients who are started on dialysis modalities in the first place is low [42]. In addition, it is notable that rate of decline in RKF in the whole cohort was significantly slower than what has been reported in observational studies previously [43] and that the proportion who progressed to anuria was low in comparison to previous evidence [44]. This was likely due to regular, detailed and checklist-driven clinical assessments of fluid balance, which were mandated in the trial protocol for both the control and intervention groups. The trial investigators hypothesise that this translated to improved accuracy in setting post-dialysis target weight and, therefore, avoidance of intravascular volume depletion and subsequent improved preservation of RKF [33]. It is possible that an association may be observed in the presence of more rapid decline in RKF and higher rates of progression to anuria. Notably, the proportion of participants reporting symptoms of dialysis-related volume depletion and fluid overload was lower than in previous reports [33]. Preservation of RKF, as well as good adherence to fluid restrictions in this highly engaged clinical trial cohort, may have mitigated the size and frequency of haemodynamic shifts and that this is one mechanism by which cognitive performance was preserved in this sample.

Our findings question the hypothesis that lower dialysate temperatures are associated with slower decline in cognition compared to higher temperatures, at least in incident haemodialysis patients [28,45]. Evidence based on outcomes in non-cardiac surgery suggests that cooling at the level of the microcirculation may induce adrenergic and metabolic changes which disrupt tissue oxygen supply and demand [46]. In addition, whilst clearance of urea does not appear to be effected by dialysate temperature, removal of toxins with larger molecular weight may be impacted [47,48]. This suggests that whilst there may be a benefit to using cooler dialysate amongst patients who are prone to intradialytic hypotension, it may have a negative impact on those with a more normal cardiovascular response to dialysis. This finding is in keeping with results from the myTEMP trial, which did not find any effect of adopting a centre-wide policy of dialysate cooling on cardiovascular outcomes, intradialytic hypotension or patient survival [49], and supports an individualised approach to setting dialysate temperature. Of interest, in the analysis of association between practice patterns and fluid status in BISTRO there was a suggestion that very low dialysate temperatures were more problematic (participants were further from their normally hydrated weight than those dialysing at higher temperatures) [50]. Dialysate temperatures were reported at centre-level in this study. However, clinicians were free to make individual temperature adjustments and this may have resulted in misclassification of the exposure in a minority of cases.

Interdialytic weight gains in the cohort overall were modest. This likely reflects relatively good preservation of RKF. Higher interdialytic weight gains were associated with improvement in MoCA score over time amongst those with preserved left ventricular function, but worsening cognition amongst those with left ventricular failure; although the relationship between weight gain and change in cognition was less clear amongst those with mean weight gains smaller than 2 kg. It is important to caveat that the left ventricular failure group contained only 18 participants and further research may be required to confirm this finding. Our results suggest that, amongst haemodialysis patients with relatively well-preserved urine output and normal left ventricular function, higher (albeit still modest) interdialytic fluid gains may be associated with slower decline in cognition over time. Inter-dialytic weight gain is strongly associated with better nutrition in the absence of comorbidity which, given that the majority of patients in BISTRO were close to their normally hydrated weight at the end of dialysis, suggests that in this context it is an indicator of better overall health and, therefore, better cognitive performance [51,52]. This study is not able to establish the direction of causality in this case. Another possible contributory factor is that many of these participants were actually mildly hypovolaemic during the initial phase of the interdialytic period. This hypothesis is supported by evidence which suggests that urinary output is lower during the first day of the interdialytic period compared to the second and third [53]. Therefore, patients with smaller weight gains may spend a longer period of time in a state of mild hypovolaemia with consequent risks of reduced organ perfusion. It is possible that larger fluid gains are poorly tolerated in those with left ventricular dysfunction, and are far less likely to reflect nutritional health, and that this results in clinical volume overload. An association between fluid overload and CI has been reported previously [31]. Volume overload is associated with hypertension, arterial stiffness, systemic inflammation and vascular calcification in the haemodialysis population [54], all of which may impact on cognitive performance. Left ventricular failure is also likely to correlate with overall burden of cardiovascular disease, which may amplify the adverse effects of fluid overload in this group. High interdialytic weight gains also necessitate larger volumes of ultrafiltration on dialysis and left ventricular failure is a risk factor for intradialytic hypotension [55]. Large volume ultrafiltration may, be more poorly tolerated in this group leading to more frequent and profound drops in blood pressure during dialysis and increased cerebral ischaemia. There was no difference in the rate of intradialytic hypotension between those with left ventricular failure and those without and our results show no association between intradialytic hypotension and change in cognition. However, asymptomatic hypotension is common, challenging to capture and associated with cerebral ischaemia [24].

This large, prospective, multi-centre study is the first to examine the association of rate of decline in RKF with change in cognitive performance over time in an incident cohort of haemodialysis patients. Our results highlight the complexity of cognitive dysfunction in this population and that cognitive performance is likely to fluctuate. Our findings may reflect selection bias in this cohort of clinical trial participants or the effect of accurate fluid balance assessment and subsequent prolonged preservation of RKF. It is reassuring that we demonstrated no evidence of deterioration in cognitive function amongst this cohort with well-managed fluid removal strategies. Our results, therefore, add to a body of evidence highlighting the importance of preserved RKF in dialysis populations. This study is also the first to examine the association of haemodialysis treatment parameters and clinical markers of volume status with change in cognition over the first two years of haemodialysis treatment. The results suggest, firstly, that an individualised approach to setting dialysate temperature is required. Secondly, that whilst modest weight gains are well-tolerated in those with normal left ventricular function, tighter fluid balance control may be beneficial for cognitive health in highly vulnerable populations with heart failure and ESKD.

## Supporting information

S1 Table STROBE Statement—checklist of items that should be included in reports of observational studies. (DOCX)

S2 Table Baseline characteristics of the cohort by change in cognition from baseline to 12 months after dialysis start (n = 158). (DOCX)

S3 Table Baseline characteristics of the cohort by change in cognition score from baseline to 24 months after dialysis start (n = 101). (DOCX)

S4 Table Results of logistic regression models for association of rate of decline in residual kidney function with cognitive impairment (MoCA^1^ < 24) at 12 and 24 months after start of haemodialysis^2^; following multiple imputation. (DOCX)

S5 Table Association of rate of decline in residual kidney function with mean change in MoCA^1^ score at 12 and 24 months after start of haemodialysis, following multiple imputation (n = 366). (DOCX)

S6 Table Association of rate of decline in residual kidney function with mean change in MoCA^1^ score at 12 and 24 months after start of haemodialysis (n = 44)^2^ – analysis restricted to those with baseline cognitive impairment. (DOCX)
