# Supplementary material for: Rate of decline in residual kidney function and cognitive impairment in incident haemodialysis patients: A prospective, longitudinal analysis of the BISTRO trial cohort
Source: PLoS One. 2026 Jun 8;21(6):e0349109. doi: 10.1371/journal.pone.0349109 (PMC13245784; doi:10.1371/journal.pone.0349109)
Supplement: S1 Table — (DOCX) [file pone.0349109.s001.docx]

**S1**

STROBE Statement—checklist of items that should be included in reports of observational studies

|  | Item No. | Recommendation | Page  No. | Relevant text from manuscript |
| --- | --- | --- | --- | --- |
| **Title and abstract** | 1 | (*a*) Indicate the study’s design with a commonly used term in the title or the abstract | 1 | “Association of rate of decline in residual kidney function with risk of cognitive impairment in incident haemodialysis patients: A prospective, longitudinal analysis of the BISTRO trial cohort” |
|  |  | (*b*) Provide in the abstract an informative and balanced summary of what was done and what was found | 2 |  |
| Introduction | | | |  |
| Background/rationale | 2 | Explain the scientific background and rationale for the investigation being reported | 4 | “There is a paucity of data examining the association between RKF and CI in haemodialysis populations. In particular, longitudinal analyses which capture change in cognition over time are lacking. Secondly, the association between clinical markers of volume status and treatment parameters, designed to reduce haemodynamic shifts and improve clearance, with CI is poorly understood” |
| Objectives | 3 | State specific objectives, including any prespecified hypotheses | 4; 5 | “This study aims, firstly, to examine the association of rate of decline in RKF with risk of CI and change in cognition amongst incident haemodialysis patients. Secondly, it aims to investigate the association of modifiable, dialysis treatment-specific factors, including treatment parameters and markers of fluid status, with change in cognition in this population.” |
| Methods | | | |  |
| Study design | 4 | Present key elements of study design early in the paper | 5 | “This is a prospective cohort study” |
| Setting | 5 | Describe the setting, locations, and relevant dates, including periods of recruitment, exposure, follow-up, and data collection | 5; 6 | “Recruitment took place over a 29 month period (April 2017 to October 2019) in 34 dialysis units across the United Kingdom (UK). “  “followed up for 24 months or until death, transplantation, withdrawal of dialysis or patient choice to leave the study” |
| Participants | 6 | (*a*) *Cohort study*—Give the eligibility criteria, and the sources and methods of selection of participants. Describe methods of follow-up  *Case-control study*—Give the eligibility criteria, and the sources and methods of case ascertainment and control selection. Give the rationale for the choice of cases and controls  *Cross-sectional study*—Give the eligibility criteria, and the sources and methods of selection of participants | 5; 6 | “Potential participants were identified using local processes in each unit^33^. Adult patients (> 18 years of age) who were within three months of commencing centre-based, maintenance haemodialysis for ESKD were eligible for inclusion.” |
|  |  | (*b*) *Cohort study*—For matched studies, give matching criteria and number of exposed and unexposed  *Case-control study*—For matched studies, give matching criteria and the number of controls per case |  |  |
| Variables | 7 | Clearly define all outcomes, exposures, predictors, potential confounders, and effect modifiers. Give diagnostic criteria, if applicable | 6; 7 |  |
| Data sources/ measurement | 8* | For each variable of interest, give sources of data and details of methods of assessment (measurement). Describe comparability of assessment methods if there is more than one group | 6 |  |
| Bias | 9 | Describe any efforts to address potential sources of bias |  |  |
| Study size | 10 | Explain how the study size was arrived at |  |  |

Continued on next page

| Quantitative variables | 11 | Explain how quantitative variables were handled in the analyses. If applicable, describe which groupings were chosen and why | 6 |  |
| --- | --- | --- | --- | --- |
| Statistical methods | 12 | (*a*) Describe all statistical methods, including those used to control for confounding | 7; 8 | “Exposures of interest were initially modelled separately to assess for confounding by ethnicity or comorbidities” |
|  |  | (*b*) Describe any methods used to examine subgroups and interactions | 8 | “Interaction terms were added to each model in turn, in order to assess for effect modification by comorbidities. “ |
|  |  | (*c*) Explain how missing data were addressed | 7 | “a complete case analysis was used.” |
|  |  | (*d*) *Cohort study*—If applicable, explain how loss to follow-up was addressed  *Case-control study*—If applicable, explain how matching of cases and controls was addressed  *Cross-sectional study*—If applicable, describe analytical methods taking account of sampling strategy |  |  |
|  |  | (*e*) Describe any sensitivity analyses | 8, 9 | “Given that there was some missing data, both of these analyses were repeated using multiple imputation as a sensitivity analysis. MoCA scores at 12 and 24 months were imputed only for those who did not die or receive a transplant and 50 imputations were run” |
| Results | | | | |
| Participants | 13* | (a) Report numbers of individuals at each stage of study—eg numbers potentially eligible, examined for eligibility, confirmed eligible, included in the study, completing follow-up, and analysed | 8 | “The cohort included 435 participants. Recruitment to the BISTRO study, including the number of exclusions and withdrawals, has been described previously” – see manuscript page 8 and also references” |
|  |  | (b) Give reasons for non-participation at each stage | 8 | “In addition, 32 (7.4%) died and 61 (14.0%) received a kidney transplant.” |
|  |  | (c) Consider use of a flow diagram |  |  |
| Descriptive data | 14* | (a) Give characteristics of study participants (eg demographic, clinical, social) and information on exposures and potential confounders | 8 | See also Table 1 |
|  |  | (b) Indicate number of participants with missing data for each variable of interest |  | See Table 1 |
|  |  | (c) *Cohort study*—Summarise follow-up time (eg, average and total amount) | 8 | “Median follow up was 13 months” |
| Outcome data | 15* | *Cohort study*—Report numbers of outcome events or summary measures over time | 8 | *“*The trial endpoint of anuria was met by 72 participants (16.6%) and follow up for patient reported outcomes continued in this group. In total, 122 participants withdrew from the trial (28%), 32 (7.4%) died and 61 (14.0%) received a kidney transplant”  See also Table 2 |
|  |  | *Case-control study—*Report numbers in each exposure category, or summary measures of exposure |  |  |
|  |  | *Cross-sectional study—*Report numbers of outcome events or summary measures |  |  |
| Main results | 16 | (*a*) Give unadjusted estimates and, if applicable, confounder-adjusted estimates and their precision (eg, 95% confidence interval). Make clear which confounders were adjusted for and why they were included | 8; 9 | See Tables 3, 4, 5 and 6 |
|  |  | (*b*) Report category boundaries when continuous variables were categorized |  | See Tables 5 and 6 |
|  |  | (*c*) If relevant, consider translating estimates of relative risk into absolute risk for a meaningful time period |  |  |

Continued on next page

| Other analyses | 17 | Report other analyses done—eg analyses of subgroups and interactions, and sensitivity analyses | 8; 9 | See Table 6 |
| --- | --- | --- | --- | --- |
| Discussion | | | | |
| Key results | 18 | Summarise key results with reference to study objectives | 10 | “CI was common in this cohort of incident haemodialysis patients, with a prevalence of 29.1% at baseline. We did not find evidence for rate of decline in RKF being associated with risk of developing CI at 12 and 24 months after start of maintenance dialysis treatment, nor with mean change in MoCA score.” |
| Limitations | 19 | Discuss limitations of the study, taking into account sources of potential bias or imprecision. Discuss both direction and magnitude of any potential bias | 10, 11 | “findings may be affected by selection bias”  “Secondly, whilst the overall sample size is large (even after accounting for drop-out) there was a significant amount of missing data, exacerbated by the impact of COVID-19 on trial procedures. Investigators were instructed to encourage the completion of patient reported outcome measures but not be forceful, and unwillingness to complete them was not a reason for trial withdrawal by the research nurses. This particularly affected MoCA scores at the 12 and 24 month timepoints, resulting in a loss of power” |
| Interpretation | 20 | Give a cautious overall interpretation of results considering objectives, limitations, multiplicity of analyses, results from similar studies, and other relevant evidence | 10; 11; 12 |  |
| Generalisability | 21 | Discuss the generalisability (external validity) of the study results | 12 | “This may reflect selection bias in this cohort of clinical trial participants or the effect of improved accuracy in assessing fluid balance and subsequent prolonged preservation of RKF. There is a need for further studies, incorporating a longer follow up time and a cohort representative of the haemodialysis population, to better understand the association between RKF and cognition.” |
| Other information | |  | | |
| Funding | 22 | Give the source of funding and the role of the funders for the present study and, if applicable, for the original study on which the present article is based | 14 | “The BISTRO Trial (ISCCTN number: 11342007) was funded by the National Institute for Health Research, (UK), Health Technology Assessment Programme under assigned grant number: 14/216/01” |

*Give information separately for cases and controls in case-control studies and, if applicable, for exposed and unexposed groups in cohort and cross-sectional studies.

**Note:** An Explanation and Elaboration article discusses each checklist item and gives methodological background and published examples of transparent reporting. The STROBE checklist is best used in conjunction with this article (freely available on the Web sites of PLoS Medicine at http://www.plosmedicine.org/, Annals of Internal Medicine at http://www.annals.org/, and Epidemiology at http://www.epidem.com/). Information on the STROBE Initiative is available at [www.strobe-statement.org](http://www.strobe-statement.org).
