# Supplementary material for: Rate of decline in residual kidney function and cognitive impairment in incident haemodialysis patients: A prospective, longitudinal analysis of the BISTRO trial cohort
Source: PLoS One. 2026 Jun 8;21(6):e0349109. doi: 10.1371/journal.pone.0349109 (PMC13245784; doi:10.1371/journal.pone.0349109)
Supplement: S2 Table — (DOCX) [file pone.0349109.s002.docx]

**S2 Table**

| Baseline characteristics of the cohort by change in cognition from baseline to 12 months after dialysis start (n = 158) | | | |  |
| --- | --- | --- | --- | --- |
|  | **Improved MoCA score (n = 68)** | **No change in MoCA score (n = 23)** | **Worsening MoCA score**  **(n = 67)** | **P – value** |
| Age (years) at study entry, mean (SD) | 62.3 (13.0) | 64.6 (13.8) | 63.7 (13.1) | 0.711 |
| Sex, n (%) |  | | | |
| Male | 53 (77.9) | 16 (69.6) | 40 (59.7) | 0.051 |
| Female | 14 (20.6) | 7 (38.4) | 27 (40.3) |  |
| Missing | 1 (1.5) |  |  |  |
| Comorbidities, n (%) |  | | | |
| Diabetes Mellitus | 24 (35.3) | 12 (52.2) | 32 (47.8) | 0.217 |
| Ischaemic heart disease | 11 (16.2) | 7 (30.4) | 13 (19.4) | 0.330 |
| Peripheral vascular disease | 6 (8.8) | 3 (13.04) | 10 (14.9) | 0.545 |
| Left ventricular heart failure | 5 (7.4) | 2 (8.7) | 8 (11.9) | 0.666 |
| Treatment type, n (%) |  | | | |
| Haemodialysis | 52 (76.5) | 17 (73.9) | 48 (71.6) | 0.815 |
| Haemodiafiltration | 16 (23.5) | 6 (26.1) | 19 (28.4) |  |
| Baseline pre-dialysis blood pressure (mmHg), mean (SD) |  |  |  |  |
| Systolic | 150.6 (15.2) | 148.2 (14.4) | 150.0 (18.6) | 0.827 |
| Diastolic | 79.6 (12.4) | 71.2 (13.3) | 74.3 (12.1) | **0.007** |
| Baseline post-dialysis blood pressure (mmHg), mean (SD) |  |  |  |  |
| Systolic | 145.1 (20.1) | 139.6 (13.9) | 143.9 (19.1) | 0.485 |
| Diastolic | 77.7 (15.7) | 72.2 (16.9) | 72.5 (13.2) | 0.09 |
| Baseline measured GFR (ml/min/1.73m^2^), mean (SD) | **n = 63** | **n = 23** | **n = 57** |  |
|  | 4.8 (2.2) | 5.1 (3.7) | 5.1 (2.8) | 0.753 |
| Baseline interdialytic urine output (ml), n (%) |  |  |  |  |
| ≤ 1500 | 38 (55.9) | 14 (60.9) | 29 (43.3) | 0.208 |
| > 1500 | 30 (44.1) | 9 (39.3) | 38 (56.7) |  |
| Mean interdialytic weight gain (kg), n (%) |  |  |  |  |
| < 0.5 | 9 (13.2) | 4 (17.4) | 10 (14.9) | 0.621 |
| 0.5 – 0.99 | 10 (14.7) | 4 (17.4) | 11 (16.4) |  |
| 1.0 – 1.49 | 19 (27.9) | 3 (13.0) | 17 (25.4) |  |
| 1.5 – 2.0 | 14 (20.6) | 5 (1.7) | 20 (29.9) |  |
| >2.0 | 16 (23.5) | 7 (30.4) | 9 (13.4) |  |
