# Supplementary material for: Rate of decline in residual kidney function and cognitive impairment in incident haemodialysis patients: A prospective, longitudinal analysis of the BISTRO trial cohort
Source: PLoS One. 2026 Jun 8;21(6):e0349109. doi: 10.1371/journal.pone.0349109 (PMC13245784; doi:10.1371/journal.pone.0349109)
Supplement: S3 Table — (DOCX) [file pone.0349109.s003.docx]

**S3 Table**

| Baseline characteristics of the cohort by change in cognition score from baseline to 24 months after dialysis start (n = 101) | | | |  |
| --- | --- | --- | --- | --- |
|  | **Improved MoCA score (n = 34)** | **No change in MoCA score (n = 18)** | **Worsening MoCA score**  **(n = 49)** | **P – value** |
| Age (years) at study entry, mean (SD) | 61.9 (13.9) | 60.8 (17.1) | 65.4 (11.4) | 0.342 |
| Sex, n (%) |  | | | |
| Male | 22 (64.7) | 12 (66.7) | 36 (73.5) | 0.759 |
| Female | 11 (32.4) | 6 (33.3) | 13 (26.5) |  |
| Missing | 1 (2.9) |  |  |  |
| Comorbidities, n (%) |  | | | |
| Diabetes Mellitus | 11 (32.2) | 9 (50) | 25 (51.0) | 0.213 |
| Ischaemic heart disease | 8 (23.5) | 3 (16.7) | 17 (34.7) | 0.275 |
| Peripheral vascular disease | 4 (11.8) | 1 (5.6) | 7 (14.3) | 0.619 |
| Left ventricular heart failure | 4 (11.8) | 4 (22.2) | 2 (4.1) | 0.08 |
| Treatment type, n (%) |  | | | |
| Haemodialysis | 29 (85.3) | 16 (88.9) | 37 (75.5) | 0.348 |
| Haemodiafiltration | 5 (14.7) | 2 (11.1) | 12 (24.5) |  |
| Baseline pre-dialysis blood pressure (mmHg), mean (SD) |  |  |  |  |
| Systolic | 143.4 (13.7) | 150.6 (16.2) | 150.0 (17.1) | 0.128 |
| Diastolic | 74.7 (11.4) | 76.1 (9.5) | 74.8 (10.1) | 0.887 |
| Baseline post-dialysis blood pressure (mmHg), mean (SD) |  |  |  |  |
| Systolic | 139.1 (18.9) | 138.9 (16.1) | 140.2 (14.9) | 0.937 |
| Diastolic | 75.0 (19.6) | 72.7 (10.0) | 73.0 (9.6) | 0.769 |
| Baseline measured GFR (ml/min/1.73m^2^), mean (SD) | n = 34 | n = 17 | n = 46 |  |
|  | 5.0 (1.9) | 6.5 (4.1) | 5.2 (2.3) | 0.135 |
| Baseline interdialytic urine output (ml), n (%) |  |  |  |  |
| ≤ 1500 | 17 (50) | 9 (50) | 21 (42.9) | 0.772 |
| > 1500 | 17 (50) | 9 (50) | 28 (57.1) |  |
| Mean interdialytic weight gain (kg), n (%) |  |  |  |  |
| < 0.5 | 7 (20.6) | 2 (11.1) | 7 (14.3) | 0.715 |
| 0.5 – 0.99 | 7 (20.6) | 2 (11.1) | 5 (10.2) |  |
| 1.0 – 1.49 | 6 (17.7) | 6 (33.3) | 14 (28.6) |  |
| 1.5 – 2.0 | 7 (20.6) | 5 (27.8) | 14 (28.6) |  |
| >2.0 | 6 (17.7) | 3 (16.7) | 9 (18.4) |  |
