# Supplementary material for: Rate of decline in residual kidney function and cognitive impairment in incident haemodialysis patients: A prospective, longitudinal analysis of the BISTRO trial cohort
Source: PLoS One. 2026 Jun 8;21(6):e0349109. doi: 10.1371/journal.pone.0349109 (PMC13245784; doi:10.1371/journal.pone.0349109)
Supplement: S4 Table — (DOCX) [file pone.0349109.s004.docx]

**S4 Table**

| **Results of logistic regression models for association of rate of decline in residual kidney function with cognitive impairment (MoCA^1^ < 24) at 12 and 24 months after start of haemodialysis^2^ ; following multiple imputation.** | | |
| --- | --- | --- |
|  | **Odds ratio at 12 months; adjusted for age and sex (n = 143)** | **Odds ratio at 24 months; adjusted for age and sex (n = 201)** |
| **Rate of decline in eGFR (per 1ml/min/1.72m^2^/month increase)** | 1.05 (0.13 – 8.39) | 1.09 (0.33 – 3.64) |
| **Age (per 1 year increase)** | 1.03 (0.98 – 1.08) | 1.04 (0.99 – 1.08) |
| **Female sex (baseline male sex)** | 1.31 (0.34 – 5.04) | 1.90 (0.62 – 5.8) |

1 Montreal Cognitive Assessment

2 Results presented as odds ratio (95% confidence interval)
