# Supplementary material for: Rate of decline in residual kidney function and cognitive impairment in incident haemodialysis patients: A prospective, longitudinal analysis of the BISTRO trial cohort
Source: PLoS One. 2026 Jun 8;21(6):e0349109. doi: 10.1371/journal.pone.0349109 (PMC13245784; doi:10.1371/journal.pone.0349109)
Supplement: S5 Table — (DOCX) [file pone.0349109.s005.docx]

**S5 Table**

| **Association of rate of decline in residual kidney function with mean change in MoCA^1^ score at 12 and 24 months after start of haemodialysis, following multiple imputation (n = 366)^2^** | | |
| --- | --- | --- |
|  | **Unadjusted mean change in MoCA score** | **Mean change in MoCA score; adjusted for age and sex** |
| **Time (per year from start of haemodialysis)** | 0.07 (-1.22 – 1.36) | 0.04 (-1.25 – 1.33) |
| **Change in eGFR (per ml/min/1.73m^2^/month)** | -0.29 (-1.41 – 0.82) | -0.25 (-1.36 – 0.87) |
| **Age at start of haemodialysis (per year)** | - | **-**0.01 (-0.05 – 0.02) |
| **Female sex** | - | 0.38 (-0.63 – 1.39) |

1 Montreal Cognitive Assessment

2 Results presented as mean 1 year change in MoCA score (95% confidence interval)
