# Supplementary material for: Rate of decline in residual kidney function and cognitive impairment in incident haemodialysis patients: A prospective, longitudinal analysis of the BISTRO trial cohort
Source: PLoS One. 2026 Jun 8;21(6):e0349109. doi: 10.1371/journal.pone.0349109 (PMC13245784; doi:10.1371/journal.pone.0349109)
Supplement: S6 Table — (DOCX) [file pone.0349109.s006.docx]

**S6 Table**

| **Association of rate of decline in residual kidney function with mean change in MoCA^1^ score at 12 and 24 months after start of haemodialysis (n = 44)^2^ – analysis restricted to those with baseline cognitive impairment** | | |
| --- | --- | --- |
|  | **Unadjusted mean change in MoCA score** | **Mean change in MoCA score; adjusted for age and sex** |
| **Time (per year from start of haemodialysis)** | -0.46 (-2.55 – 0.87) | -0.32 (-2.44 – 1.80) |
| **Change in eGFR (per ml/min/1.73m^2^/month)** | -2.21 (-6.20 – 1.79) | -2.10 (-6.27 – 2.07) |
| **Age at start of haemodialysis (per year)** | - | -0.06 (-0.11 – 0.00) |
| **Female sex** | - | -0.40 (-2.21 – 1.42) |

1 Montreal Cognitive Assessment

2 Results presented as mean 1 year change in MoCA score (95% confidence interval)
